# Gestational Exposure to Synthetic Steroid Hormones Impaired Sperm Quantity and Quality in Wistar Rats

**DOI:** 10.1155/2020/1814867

**Published:** 2020-01-25

**Authors:** Samy Ismail Ahmed, Aamir Magzoub, Mohammed Saeed Zayed Al-Ayed, Gamal Ali Attia, Basel A. Abdel-Wahab, Masood M. Khateeb, Asim M. Abdalla, Heitham M. Mohammed, Aymen N. Eldeen A. Elkareem, Ali Gadkarim A. Salih

**Affiliations:** ^1^Department of Anatomy, College of Medicine, Najran University, Najran, Saudi Arabia; ^2^Department of Physiology, College of Medicine, Najran University, Najran, Saudi Arabia; ^3^Department of Paediatrics, College of Medicine, Najran University, Najran, Saudi Arabia; ^4^Department of Anatomy, College of Medicine, Assiut University, Assiut, Egypt; ^5^Department of Pharmacology, College of Medicine, Assiut University, Assiut, Egypt; ^6^Department of Pharmacology, College of Medicine, Najran University, Najran, Saudi Arabia; ^7^Department of Anatomy, College of Medicine, King Khalid University, Abha, Saudi Arabia

## Abstract

This study was designed to investigate the effect of prenatal exposure to synthetic sex steroid on sperm quantity and quality, relative testicular and epididymal weights, and reproductive hormones level in adult Wistar rats. Forty male Wistar rats were divided into two groups: a test group (*n* = 20) that included mature rats that were born to dams exposed to gestational treatment with hydroxyprogesterone and a control group (*n* = 20) that included mature rats born to untreated dams. Compared to the control group, the test group showed a significant reduction in the sperm count, viability and motility, relative testicular and epididymal weights together with increased abnormal spermatozoa (*p* < 0.001). The reproductive hormonal assay revealed significantly lower serum testosterone and higher levels of FSH and LH among the test groups compared to the control (*p* < 0.05 for all). Prenatal exposure to synthetic progesterone negatively affected sperm production and function, relative testicular and epididymal weights, and reproductive hormone levels.

## 1. Introduction

Recently, and with a significant progressive increase in the incidence of human infertility and testicular disorders, considerable concern has been expressed over the possibility that prenatal exposure to excess synthetic sex hormones and steroids may adversely affect male reproduction in both animals and humans [1, 2].

Clinical and experimental reports support the hypothesis that the fetal origin of testicular disorders is associated with exposure to endocrine disrupters. New data reported that prenatal exposure to such hormones is likely to cause testicular developmental disorders and, consequently, induce infertility [3–5]. Nevertheless, prenatal exposure to progesterone was reported to induce irreversible testicular growth alteration, reduced sperm production, and suppressed steroidogenesis [5, 6].

Hydroxyprogesterone is synthetic progesterone prescribed traditionally for many obstetric and nonobstetric conditions. In obstetrics, the hormone is used for the treatment of abnormal uterine bleeding, threatened miscarriages during the first trimester, primary infertility cases, preterm delivery prevention, and to prevent and treat abnormal endometrial thickening (endometrial hyperplasia). Nonobstetric uses include treatment of mammary pain in women with noncancerous breast disease and as a topical treatment in certain skin diseases [7–11].

Experimental studies showed that exposure to such drugs during the embryonic period of development that is marked as a critical period might significantly decrease the epididymal sperm count and sperm motility in rats. These abnormalities may result from alteration of the normal endocrine system function of the animal and interferences with synthesis, secretion, transport, metabolism, binding, and elimination of natural blood hormones that are responsible for homeostasis, reproduction, and developmental process [11, 12].

Following exposure to hydroxyprogesterone, recent evidence revealed abnormal morphometric and histological changes, including a reduction in sperm count, damaged seminiferous tubules, and decreased testicular and epididymal weights [7, 8, 11].

Although many experimental protocols extensively studied the impacts of prenatal exposure to estrogenic steroids on male and female reproduction, data are scarce concerning the male reproductive changes following prenatal exposure to progestogens. In this context, the present study was conducted to further explore the effects of prenatal exposure to hydroxyprogesterone on sperm quantity, sperm quality, relative testicular and epididymal weights, and reproductive hormone level using an experimental approach.

## 2. Material and Methods

### 2.1. Chemicals

Synthetic progesterone, hydroxyprogesterone caproate (Hydroxyprogesterone; Schering AG; Germany. Trade name: Proluton Depo ®), is available in an oily solution diluted by pure Spanish olive oil (1 : 4 ml). It was obtained from Najran Maternity Hospital.

### 2.2. Animals

Eight to ten weeks' old, pregnant females were divided into two groups and kept separate away from any stress in sterilized polypropylene cages (90 cm × 45 cm × 15 cm) lined with woody husk at 12 : 12 h light/dark cycle, (28 ± 7) °C temperature, fed on a commercial pellet and offered water ad libitum. Group one (*n* = 10) served as a control, and group two as a test group (*n* = 10). The females in the test group were subcutaneously injected with 10 mg/kg of hydroxyprogesterone on the 1st, 7th, and 14th day of gestation. The females in the control group were injected with a similar dose of a placebo (pure Spanish olive oil).

Twenty male puppies born to each of the test and control groups were randomly selected and allowed to grow for 90 days where they reached maturity. The test group included male rats that were born to dams treated with synthetic progesterone during pregnancy, whereas those who born to untreated dams served as a control (*n* = 20 in each group). The dams' body weights were taken daily to adjust the dose [8, 11, 13]. The dose used in this study was in the range of regular clinical use and the administrative schedule is similar to humans during pregnancy [11].

### 2.3. Tissue Collection and Preparation

Rats were anesthetized with chloroform, sacrificed by cervical dislocation, and blood samples were collected from each animal through cardiac puncture using a 3 ml syringe and placed in 5 ml plain blood collection tube to determine the serum levels of testosterone, luteinizing, and follicle-stimulating hormones.

The right testis and epididymis of each rat were quickly removed and cleaned from surrounding connective tissues and then weighed. The mid-to-distal region of the epididymis was perforated in a Petri dish by 3 mL needle. The oozed sperms containing fluid was then diluted in 1 mL physiological saline solution (0.9% NaCl). The apparent sperm suspension was gently mixed and kept at 37°C for 5 minutes to allow for the dispersion of sperms in the medium [4, 12]. After thorough mixing, the sperm suspension was used to assess the sperm count, motility, viability, and morphology. Later, relative testis and epididymis weights were calculated per final body weight [11, 14].

### 2.4. Semen Analysis

#### 2.4.1. Sperm Counts

A drop of the diluted semen (1 : 20), thoroughly mixed, was transferred to a Neubauer hemocytometer using a micropipette, and a cover glass overlaid. The total number of the sperms was then observed and counted under a Carl Zeiss (Germany) Axio 2 Plus microscope at ×100 magnification. Sperms were counted in five small squares of the large central square, each square consisting of 16 smaller squares. Therefore, sperm concentration was expressed in terms of sperm X 10^6^/ml. Two samples from each epididymis were examined, and the average counts were scheduled [12, 15–17].

#### 2.4.2. Sperm Motility

The sperm motility was assayed microscopically within 5 minutes following their isolation from cauda epididymis at 37°C. A drop of sperm suspension was placed on a Neubauer hemocytometer using a micropipette and then observed under a Carl Zeiss (Germany) Axio 2 Plus microscope at ×100 magnification. Sperm with any of the different motility types was recorded as motile sperm. Sperm motility was expressed as a percentage of motile sperm of the 200 sperm counted in 10 randomly selected fields for each rat [4, 11].

For each animal, two separate hanging drops were prepared to obtain the average.

#### 2.4.3. Sperm Viability

This technique was used to differentiate between live and dead sperms. A drop of the diluted semen was transferred to an Eppendorf tube (1 mL) containing one drop of 1% Eosin stain. The contents were mixed gently, left for 5 minutes at 37°C, and about 10 *μ*L of the sample was then observed under a Carl Zeiss (Germany) Axio 2 Plus microscope at ×200 magnification. The head of dead spermatozoa was stained red while the live spermatozoa were unstained with Eosin. Sperm viability, expressed as a percentage of live sperms of the 200 sperms, was evaluated in 10 randomly selected fields for each rat [4, 11]. For each animal, two separate hanging drops were prepared, and two independent observers assessed the viability. The data from each animal were used to obtain the average.

#### 2.4.4. Sperm Morphology

A gently mixed drop of the sperm suspension in an Eppendorf tube (1 mL) was placed on a clean slide and gently spread to make a thin film. The film was air-dried [18] and then observed under a Carl Zeiss (Germany) Axio 2 Plus microscope using ×100 magnification. The relative percentages of abnormal sperms were counted from 10 different optical fields for each rat sample. Abnormal sperms included headless, tailless, and coiled tail [11, 13, 19]. For each animal, two separate hanging drops were prepared, and two independent observers assessed the abnormalities. The average results of each animal were obtained.

### 2.5. Serum Collection and Hormonal Assay

The blood serum was separated by centrifugation at 4,000 rpm for 5 minutes after overnight storage at 4°C and then stored at 20°C [8, 11, 12]. Specific commercially available ELISA kits, purchased from Elabscience Biotechnology Co., Ltd (Elabscience), China, were used to assess rat luteinizing hormone (LH) Catalog No: E-ELR0026, testosterone (Catalog No: E-EL-R0155) and follicle-stimulating hormone (FSH) (Catalog No: E-EL-R0391) serum level according to their manufacturer's method instructions listed on the following table (Table 1):

### 2.6. Statistical Analysis

The obtained data were analyzed using SPSS version16 (Chicago, USA) software program. Data were expressed as mean ± SD. One-way analysis of variance (ANOVA) was used to test the significant difference between different groups with the level of significance set at *p* < 0.05.

### 2.7. Ethical Approval

This study was committed to national and international ethical experimental protocols and was approved by college of medicine ethical approval committee—Najran University-KSA.

## 3. Results

### 3.1. Sperm Count

The mean epididymal sperm count per 0.1 g/epididymis of the test group was significantly lower from that of the control (*p* < 0.001) (Table 2), which reflects a clear difference in spermatozoa density between the progesterone-treated and the control groups.

### 3.2. Sperm Motility and Sperm Viability

**Figure 1:**
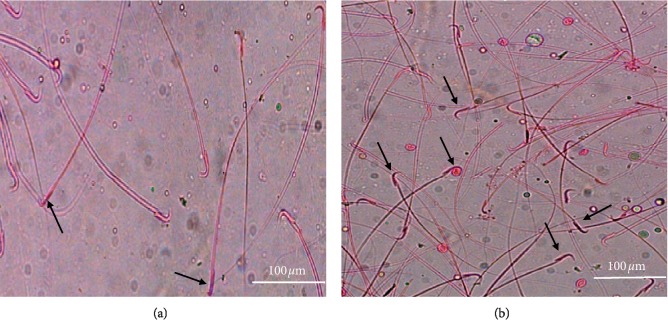
Micrographs illustrating sperm viability: control group (a), and the test group (b). Note the difference in the number of dark-stained dead sperms (yellow arrows) (×200).

The mean percentage of the sperm motility and viable spermatozoa showed a significant reduction in the test group compared to the control (*p* < 0.001) (Table 2, Figure 1).

### 3.3. Sperm Morphology

**Figure 2:**
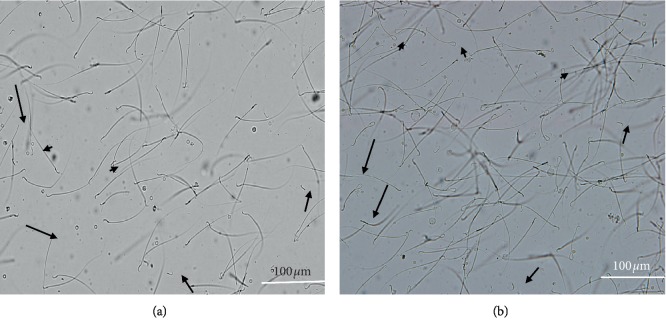
Micrographs showing sperms with abnormal morphologies including headless (long arrow), tailless (short arrow), and coiled tail (arrowhead) in the control (a) and test groups (b) (×100). Note that the number of abnormal sperms is higher in the test group compared to the control.

A significant increment in the total number of abnormal spermatozoa in the test group was observed compared to control. The mean percentages of the total and specific (headless, tailless, and coiled tail) abnormalities were significantly higher among the test group (Table 3, Figure 2).

### 3.4. Relative Testicular and Epididymal Weight

Compared with the control group, the mean percentage of relative testicular and epididymal weights of the test group showed a significant reduction (*p* < 0.01) (Table 4).

### 3.5. Hormonal Assay

Serum testosterone levels were significantly decreased (*p* < 0.001) in male rats born to dams treated with synthetic progesterone during pregnancy. However, serum FSH and LH levels were both significantly increased in the test group (Table 5).

## 4. Discussion

The present study showed interesting findings that uphold the previous studies [11, 12] in that prenatal exposure to synthetic progesterone negatively affected sperm production, sperm function, and testicular hormone levels.

The sperm count, motility, and viability were reduced significantly among the progesterone-treated (test) group compared to the control one, an effect that might be due to hormonal-induced abnormal alteration in the testicular structure and function during the embryonic period, which in turn affected sperm development and maturation. These findings were consistent with few previously reported findings that investigated the effects of prenatal exposure to progesterone on male mice and rat reproduction, respectively [11, 12]. Nevertheless, the present study boosts the fact that prenatal exposure to such a hormone induces long-term abnormalities on testicular histology, and confirmed a significant change in weights of the reproductive organs, sperm quantity and quality, serum reproductive hormone concentrations, and fertility at maturity following prenatal and neonatal rats' exposure to contraceptive compounds [20]. The same findings were reported after the gestational and lactational exposure of male mice to diethylstilbestrol [21].

On the other hand, the significant increment in the percentages of the total abnormal spermatozoa, headless, tailless, and coiled tail spermatozoa in the test group compared to the control suggested that administration of progesterone during pregnancy might also disrupt sperm quality and therefore may impair fertility [1, 20].

A significant decrease in the relative testicular and epididymal weights (*p* < 0.01) and in the test group compared to control was documented in the present study. This result might be due to germinal and somatic cell loss or hypotrophy, shrinkage of seminiferous tubules, decrease in the sperm count, and increase in the sperm abnormalities (headless and tailless) due to prenatal progesterone exposure. These findings are consistent with similar studies [5, 22]. The weight of the testis is mainly dependent on the mass of differentiated spermatogenic cells, and it has been used as a measure of spermatogenesis in rats [2]. A positive correlation between the weight of testis and the number of germ cells also was observed [8, 23].

The present study also included the effect of prenatal progesterone treatment on the reproductive hormones, namely testosterone, LH, and FSH serum levels. Compared to controls, a significant reduction in the serum testosterone level as well as a significant increase in the serum FSH and LH level were observed in the test group. Similar findings were reported in rats following prenatal exposure to hydroxyprogesterone [11, 12] as well as neonatal exposure to estrogen [24, 25]. The reduction in serum testosterone levels in the test group might be explained by a reduction in the Leydig cell count, diminished responsiveness of Leydig cells to LH, or direct inhibition of testicular steroidogenesis [5, 25]. A significant decrease in the 3b-hydroxysteroid dehydrogenase (3b-HSD) and 17b-hydroxysteroid dehydrogenase (17b-HSD) activities and steroidogenesis in the testes of mice prenatally exposed to hydroxyprogesterone was reported [6]. As testosterone is essential for the normal development of sperms in terms of different stages in the spermatogenesis process, the low hormone levels in the test group are expected to contribute to the observed reduction in the sperm count. The high serum FSH levels observed in the test group could be due to germ cell loss in the spermatogenic compartment or damage to the Sertoli cells, which is expected to decrease inhibin hormone, thereby affecting the negative feedback regulation of FSH secretion.

Similarly, the increased levels of LH, together with decreased levels of serum testosterone in the test groups, are indicative of loss of the negative feedback of testosterone on LH secretion caused by impairment of Leydig cell structure or function. Earlier studies reported that neonatal exposure to diethylstilbestrol resulted in suppression of androgen action in addition to abnormalities in the male reproductive tract. Nevertheless, the increased LH serum level delayed the onset of mesenchymal cell differentiation into Leydig cells [12, 22, 26, 27].

In conclusion, the present study showed that prenatal exposure to synthetic progesterone adversely affected sperm production and function, relative testicular and epididymal weights, and reproductive hormone levels. These findings raise a question on the safety profile of progesterone use during pregnancy, particularly in the presence of any testicular abnormality.

## Figures and Tables

**Table 1:** 

Hormone	Sensitivity	Intra-assay coefficients of variability Midrange (%)	Interassay coefficients of variability Midrange (%)
LH (mIU/mL)	0.94	4.55	5.96
FSH (ng/ml)	1.88	1.88	5.6
Testosterone (ng/ml)	0.17	6.72	6.18

**Table 2:** Mean percentage of the sperm count, motility, and viability in the control and test groups.

Parameter	Control	Test group	*p* value
Sperm count (million/ml)	(113.55 ± 10.46)	(81.72 ± 5.61)	(*p* < 0.001)
Motility (%)	(82.42 ± 6.62)	(63.65 ± 6.49)	(*p* < 0.001)
Viability (%)	(81.82 ± 6.54)	(62.90 ± 6.10)	(*p* < 0.001)

**Table 3:** Mean percentage of total and specific abnormalities in the control and test groups.

Parameter	Control group	Test group	*p* value
Total sperm abnormality (%)	(15.10 ± 1.42)	(41.16 ± 3.93)	(*p* < 0.005)
Headless	(1.42 ± .41)	(9.78 ± 1.05)	(*p* < 0.005)
Tailless	(1.91 ± .48)	(16.42 ± 2.78)	(*p* < 0.005)
Coiled tail	(2.86 ± .52)	(16.38 ± 3.89)	(*p* < 0.001)

**Table 4:** Effects of synthetic progesterone on the testicular and epididymal relative weights (g).

Parameter	Control	Test I	*p* value
Relative testicular weight	(0.011 ± 0.0002)	(0.0062 ± 0.0002)	(*p* < 0.001)
Relative epididymal weight	(0.004 ± 0.001)	(0.002 ± 0.0002)	(*p* < 0.001)

**Table 5:** Mean serum reproductive hormones (ng/ml) in the control and test groups.

Parameter	Control	Test group	*p* value
LH	(3.13 ± 0.10)	(3.9 ± 0.10)	*p* < 0.01
FSH	(3.90 ± 0.14)	(5.0 ± 0.10)	*p* < 0.01
Testosterone	(2.33 ± 0.09)	(1.04 ± 0.06)	*p* < 0.01

## Data Availability

The data used to support the findings of this study are available from the corresponding author upon request.
